# Thermoregulation in the wild boar (Sus scrofa)

**DOI:** 10.1007/s00360-023-01512-6

**Published:** 2023-09-24

**Authors:** Thomas Ruf, Sebastian G. Vetter, Johanna Painer-Gigler, Gabrielle Stalder, Claudia Bieber

**Affiliations:** 1 Department of Interdisciplinary Life Sciences, Research Institute of Wildlife Ecology, University of Veterinary Medicine, Savoyenstrasse 1, 1160 Vienna, Austria; 2 Present Address: Department for Farm Animals and Veterinary Public Health, Institute of Animal Welfare Science, University of Veterinary Medicine, Veterinärplatz 1, 1210 Vienna, Austria

**Keywords:** Thermoregulation, TNZ, Heart rate, Core–shell, Subcutaneous temperature, Mass

## Abstract

The wild boar (*Sus scrofa*) originates from warm islands but now inhabits large areas of the world, with Antarctica as the only continent not inhabited by this species. One might be tempted to think that its wide distribution results from increasing environmental temperatures. However, any effect of temperature is only indirect: Abundant availability of critical food resources can fully compensate the negative effects of cold winters on population growth. Here, we asked if temperature as a habitat factor is unimportant compared with other habitat indices, simply because wild boars are excellent thermoregulators. We found that the thermoneutral zone in summer was approximately 6–24 °C. In winter, the thermoneutral zone was lowered to 0–7 °C. The estimated increase in the heart rate and energy expenditure in the cold was less than 30% per 10 °C temperature decline. This relatively small increase of energy expenditure during cold exposure places the wild boar in the realm of arctic animals, such as the polar bear, whereas tropical mammals raise their energy expenditure several fold. The response of wild boars to high T_a_ was weak across all seasons. In the heat, wild boars avoid close contact to conspecifics and particularly use wallowing in mud or other wet substrates to cool and prevent hyperthermia. Wild boars also rely on daily cycles, especially of rhythms in subcutaneous temperature that enables them to cheaply build large core–shell gradients, which serve to lower heat loss. We argue it is predominantly this ability which allowed wild boars to inhabit most climatically diverse areas in the world.

## Introduction

The current distribution of wild boars (*Sus scrofa*) extends to most areas of the world with Antarctica being the only continent not inhabited by this species (Lewis et al. 2017). In fact, no other mammal, except for humans and their companions the house mouse (*Mus musculus*) and the Norwegian rat (*Rattus norvegicus*), has settled in as many climatically diverse areas. Its evolutionary lineage originates from islands in Southeast Asia (Philippines, Indonesia) (Chen et al. 2007), and in warm countries such as Vietnam, wild boars still thrive and pig farming is an important meat source (Thom et al. 2021). *S. scrofa* naturally expanded throughout Eurasia and into North Africa (Wehr 2021). Remarkably, now the wild boar in Europe is distributed up to 64°N. In Asia, the northern distributional limit is up to 61°N and it was reported to inhabit even the coldest climates in Siberia (Markov et al. 2019).

In fact, the wild boar is not only a widespread species but has become a pest in large areas. In many parts of Europe, densities of wild boars have been growing notably during the past few decades (e.g., Vetter et al. 2015). This increase has led to substantial damage to agricultural crops and can even cause problems in urban habitats (Geisser and Reyer 2005). Feral pigs in Australia, New Zealand, and other southern countries cause similar damage (Dexter 2003; Hampton et al. 2004). The increase in wild boar populations may be the result of the global increase in mean environmental temperatures. However, any effect of ambient temperature (T_a_) is only indirect, via food. The abundant availability of critical food resources, e.g., beech nuts, can outweigh the negative effects of cold winters on population growth of wild boar (Vetter et al. 2015).

More recently, the regions of successful settlement of wild boars were analyzed in detail based on 129 records of population densities and habitat characteristics (Lewis et al. 2017). Interestingly, this study showed a remarkable influence of both abiotic and biotic factors, such as large carnivore richness. Ambient temperature seemed to be less important but may play a role on a broad scale (Lewis et al. 2017). However, one may argue that for a species that is excellent at thermoregulation, T_a_ as a habitat factor may seem unimportant compared with other habitat indices, especially if T_a_ does not constrain population size. Yet, we are unaware if wild boars are excellent or poor thermoregulators.

Measurements of energy expenditure for thermoregulation were mostly carried out on domestic pigs that are characterized by a thin layer of sparse hairs, and this work often focused on newborn piglets that are especially temperature sensitive (reviewed in Gómez-Prado et al. 2022). In sows, the thermoneutral zone (TNZ) has been reported to be 15–20 °C (Yousef 1985). For an overview on heat stress in pigs, see Brown-Brandl et al. (2004). Measurements on actual wild boar also seem restricted to observations mostly on juveniles (Jezierski and Myrcha 1975). Part of the reason for the scarcity of metabolic data is that adult wild boars tend to destroy respirometric chambers.

We asked if wild boars are relatively cold tolerant and have a lower TNZ than domesticated pigs. In addition, we were interested in how much wild boars raise their energy expenditure for thermoregulation. Indirectly, we wanted to see if their ability to inhabit most climatically diverse areas of the world is largely due to excellent thermoregulation. We addressed these questions using heart rate measurements. We further implanted temperature loggers at different parts of the body to measure a possible temperature gradient in the body in detail.

## Material and methods

### Animals and study area

The study animals were kept in an outdoor enclosure (~ 55 ha) in the Burgenland (Austria). The study enclosure was covered with a deciduous forest, mainly Turkey oak (*Quercus cerris*) and pubescent oak (*Quercus pubescens*) and included a few meadow patches. For the present study, 13 adult females were used. Likely, all females gave birth and were lactating. We concentrated on females only because the live capture and handling of males are hampered by their large size and ferocity. Also, due to competition and high levels of aggression between males during rut, the stocking of the enclosure was strongly female biased. During the study period (12/2016–01/2019), the animal density was ~ 1 adult female/ha plus up to 20 males (total) of different ages. Due to this relatively high density, animals were supplemented with 1–1.5 kg corn/individual once a day (at 2:00–14:00 h) at two feeding areas, each ~ 40 × 20 m. The enclosure was enclosed by 2.5 m high, solid, non-transparent fencing and was closed for the public. Thus, the study site provided an environment without disturbances due to hikers, bikers or straying dogs. There were no battue hunts or other disturbances due to hunting or forest management activities during the study period in the enclosure.

Animals were trapped once a year in autumn within the feeding sites to collect data on reproductive success (collecting DNA of juveniles) and body condition of females and to separate some of them for implantation/explantation of loggers. While feeding, we closed the access gates and released the boar one by one through a wooden corridor back into the enclosure. While in the wooden corridor, we recorded the body mass of each individual (Gallagher SmartScale® 500, Groningen, Netherlands). Due to management reasons, the juveniles (born in spring) were removed from the enclosure during this procedure.

### Implantation of temperature and heart rate loggers

**Fig. 1:**
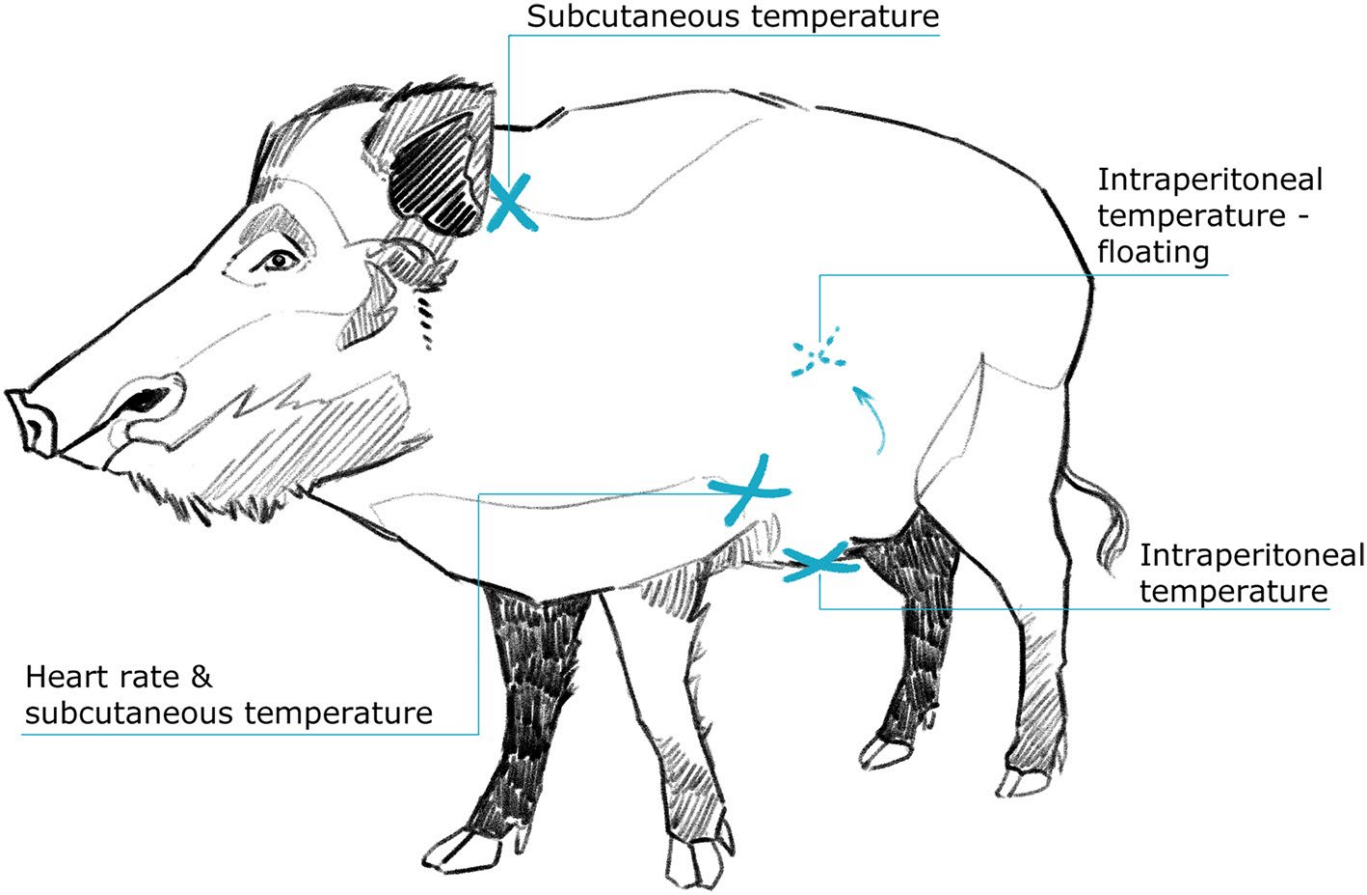
Location of the implantation of loggers on wild boar. Each female investigated had a least two and up to 5 loggers (heart and temperature) implanted

We implanted a heart rate and temperature logger (HR&T; DST centi-HRT, Star-Oddi, Gardabaer, Iceland, and custom built at the Research Institute of Wildlife Ecology, Vienna, Austria, Fig. 1). In ten females, we implanted heart rate and temperature loggers at the sternum as well as temperature loggers at the neck. Nine of these females also received intraperitoneal wall temperature loggers, and in the tenth animal, the logger was internally floating (ruptured from the intraperitoneal wall). Three additional females were implanted with intraperitoneal-floating and wall temperature loggers. Two of these three also had subcutaneous neck loggers. All 13 animals underwent surgery in October/November 2016 and 2017 as adults (females aged 5 and 6 years). Logger records lasted up to a little more than one year. All details about surgery techniques and anesthesia protocols were provided before (Ruf et al. 2021). Explantations were carried out approximately one year after implantations. The last explantation was carried out in January 2019. Mean body mass at date of implantation for all females was 71.8 ± 15.5 kg. Body mass was not determined during HR measurements and too rarely to establish an allometry.

The HR&T logger (Fig. 1) sampled data at 12 min intervals without specific limits to maximum and minimum. We did not further validate records. It was positioned subcutaneously, in proximity to the heart on the lateral rib cage, behind the moving area of the elbow, to avoid rubbing, or inserted and tethered into the ventral subperitoneal space caudal of the xiphoid process of the sternum.

Self-built temperature loggers were covered with inert surgical wax and had a weight of ~ 8 g. Time interval of recording was also adjusted to 12 min, the accuracy 0.01 °C. One of the temperature loggers had an especially flat shape (3.4 × 1.9 × 0.5 cm) to fit smoothly into the subcutaneous neck region (*n* = 10). The second temperature logger (diameter = 2.1 cm and height = 1.2 cm) was placed into the intraperitoneal cavity, either tethered at the linea alba (*n* = 12), or freely floating in the cavity (*n* = 4), usually found between two lobes of the liver.

### Activity data

To record the activity (ear-tag movements) of animals, a telemetry system (Smartbow System, Zoetis, New Jersey, USA) was installed around the two neighboring feeding areas and two close water ponds in the enclosure. The system consisted of a central solar power and computing station and ten receivers located at the height of 2–3 m. Part of the system were ear-tags (34 g; 52 × 36 × 17 mm, *n* = 13). The accelerometer (located inside ear-tags) measured triaxial acceleration (x, y, z). As an estimate of movements, we computed the total acceleration vector from sqrt (*x*^2^ + *y*^2^ + *z*^2^).

### Climate

We recorded ambient temperature (T_a_) and black-bulb temperature (T_ab_) at 2 m height directly at the study site (Vantage Pro 2 with black-bulb extension, Davis Instruments, Hayward, USA).

### Data analysis

We did not assess the influence of environmental conditions in different years, due to logger failures and thus scarcity of heart rates, and all data were pooled for different years (with similar conditions and food available year- round). Together, we analyzed a little more than 0.5 million data sets (with equal contributions from times, heart rates, activities and temperatures).

**Fig. 2:**
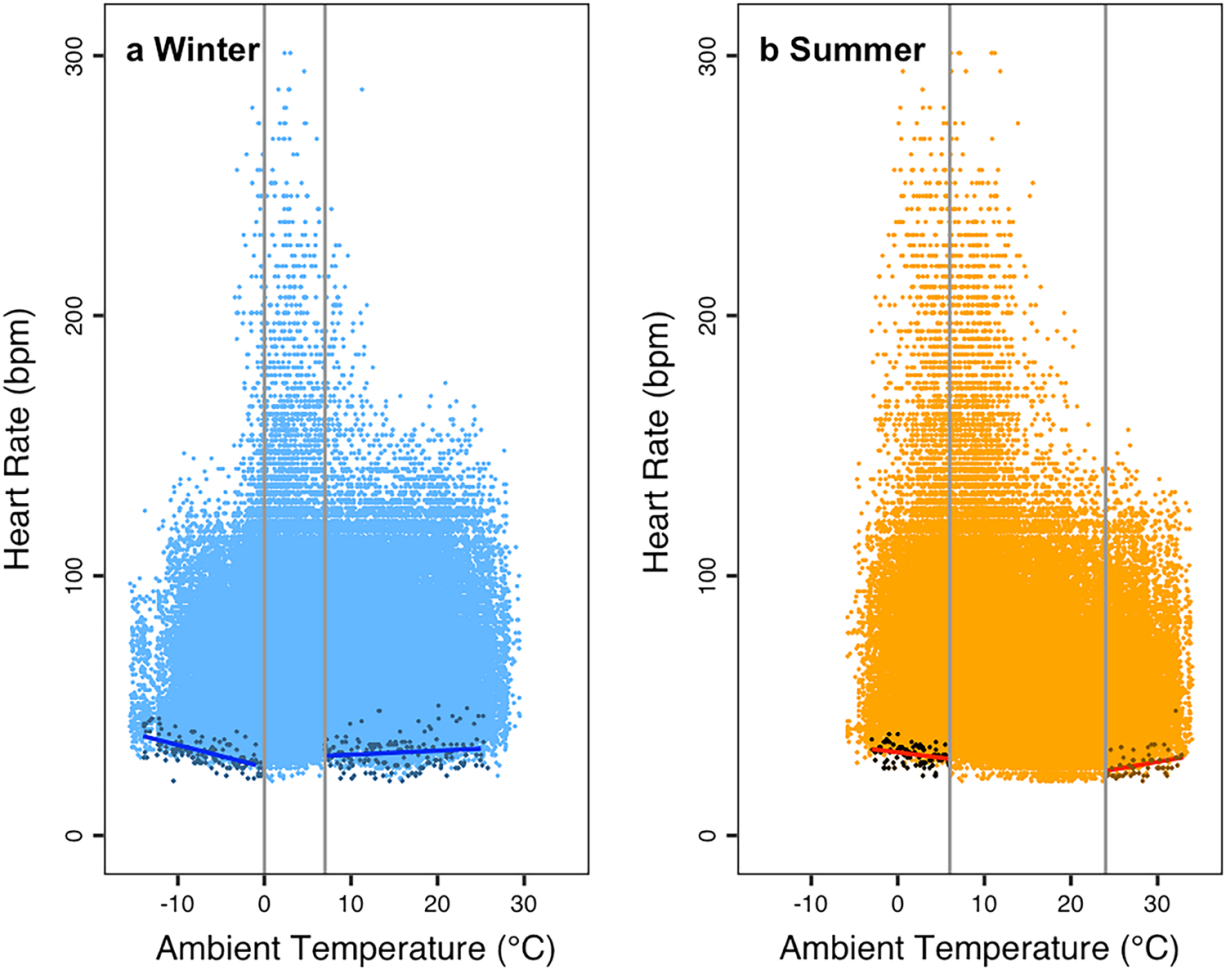
Heart rate as a function of T_a_ in wild boar during winter (**a** December–May) and summer (**b** June–November). Dots are original heart rates. The thermoneutral zone in summer was approximately 6–24 °C (vertical lines). In winter, the thermoneutral zone was approximately 0–7 °C (vertical solid gray lines). Red regression lines how increases below thermoneutrality in summer, blue lines depict winter. Linear regressions were computed using several minimum heart rates per animal and degree centigrade

To remove outliers, all initial data from recorders were subjected to the R package boxfilter that removes isolated data points (Ruf 2022). To investigate the influence of T_a_, heart rates were plotted with ggplot2 (Wickham 2016) as a function of T_a_ for meteorological summer (01 June–31 October) and winter (01 December–31 May). During warm and cold exposure in both seasons, linear regressions were computed using several minimum heart rates per animal and degree centigrade (Fig. 2). To restrict the data analysis to thermoregulation, we deleted 5 out of 495 regression points, with relatively high heart rates (> 50 bpm) when animals were apparently not at rest. The relationship between heart rate at rest and T_a_ was investigated for summer and winter separately by using the R package segmented (Muggeo 2003). This library determines best breakpoints in linear relations, in this case the critical temperatures below and above the TNZ. Suggested breakpoints were rounded to integers (in °C).

The relation between peripheral temperatures and T_a_ was investigated with general additive models (R package mgcv (Wood 2017), Figs. 2, 3). This function fits non-linear splines to the data, which are penalized for their “wiggliness”, i.e., the number of turning points in the fit. These models were all corrected for the time of day (using circular cubic splines), the random effects of individuals, and for autocorrelation (using rho and the AR.start function).

**Fig. 3:**
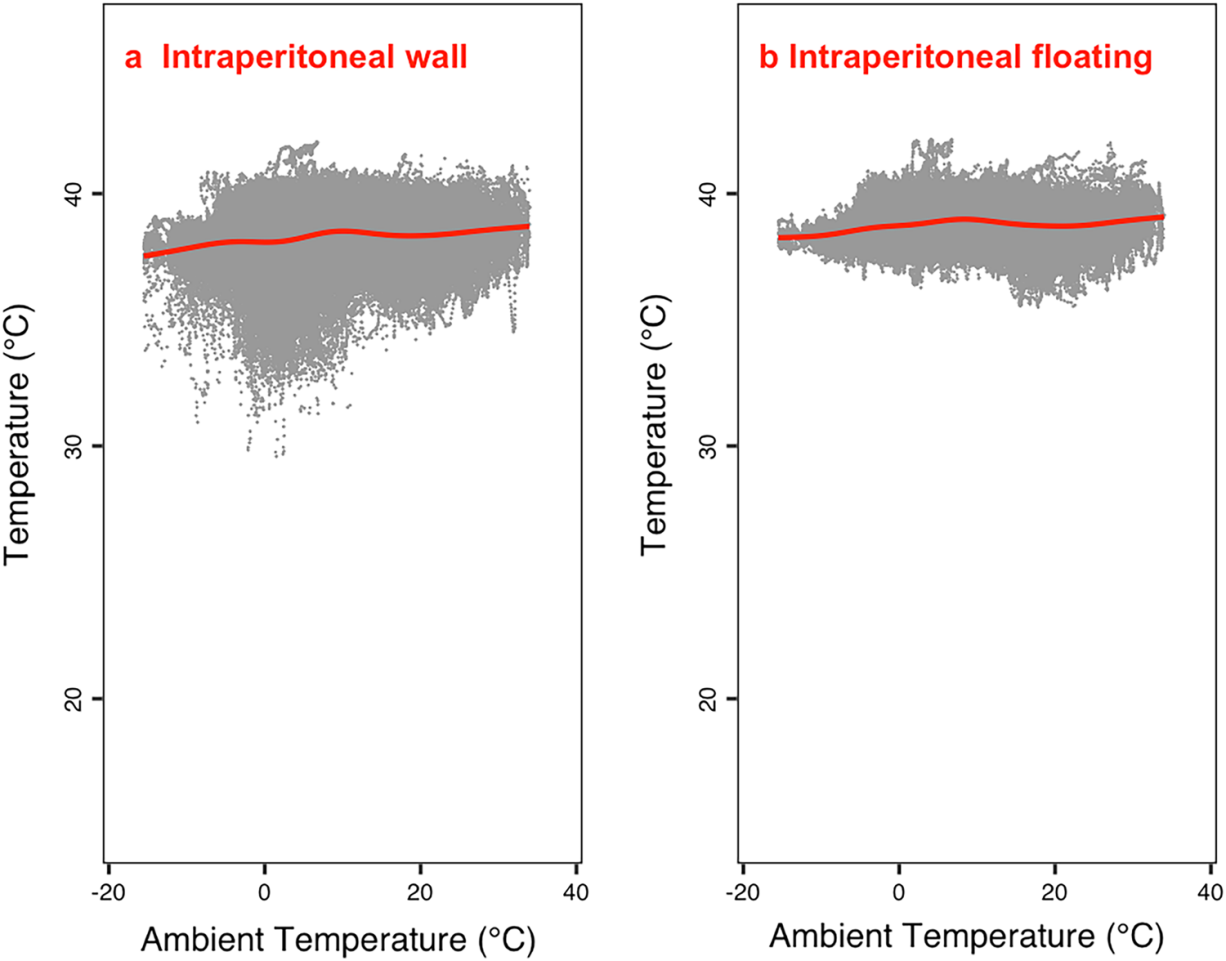
Intraperitoneal temperature as a function of T_a_ in wild boar. Temperature loggers measured T_b_ at 12 min intervals and were either sutured to the intraperitoneal wall (la) or free floating (found next to the liver, B). GAM models (red lines) indicated that intraperitoneal temperatures decreased with T_a_ (floating: edf (wiggliness) = 8.91, residual df = 8.99, *F* = 867.9, *P* < 0.0001***). Partial residual plots corrected for the influence of random differences between individuals. For loggers fixed to the wall, model parameters were (edf (wiggliness) = 8.92, residual df = 8.99, *F* = 1030, *P* < 0.0001***). All models were also corrected for initial autocorrelation (> 0.9)

To illustrate the effects of time of day, means ± SEM of several measured variables were shown as a function of mean hour (computed using R package lubridate (Grolemund and Wickham 2011).

## Results

The mean heart rate over the entire range of T_a_ was 66.02 ± 0.051 bpm, approximately three times the minimum heart rate per female (mean: 22.3 ± 0.53). Heart rates were highest (> 200 bpm) at T_a_ around −2–12 °C (Fig. 2). Ambient temperature ranged from −15.5 °C in winter to 34 °C in summer (Fig. 2). There was no appreciable correlation between ear-tag movements and heart rate (*r* = 0.028), not even when activity was shifted backward several time intervals (assuming a delayed response of heart rate). Thus, ear-tag movements apparently were not associated with elevated locomotion and heart rate.

In summer, minimum heart rate increased above 24 °C (*Y* = 14.87 + 0.44 × T_a_) and below 6 °C (*Y* = 31.85–0.48 × T_a_). This was calculated from a regression through minimum points per T_a_ including every animal; Fig. 2). Accordingly, the apparent thermoneutral zone in summer was approximately 6–24 °C (vertical lines). In winter, heart rate increased above 7 °C (*Y* = 29.86 + 0.12 × T_a_) and also below 0 °C (*Y* = 26.28–0.85 × T_a_) (Fig. 2). Thus, in winter, the thermoneutral zone was 0–7 °C. Wild boar increased their minimum heart rate by 13.2% in summer and 28.4% in winter per additional 10 °C at low T_a_s. At temperatures above the apparent TNZ, the increase was 25.9% in summer and merely 4.0% in winter.

**Fig. 4:**
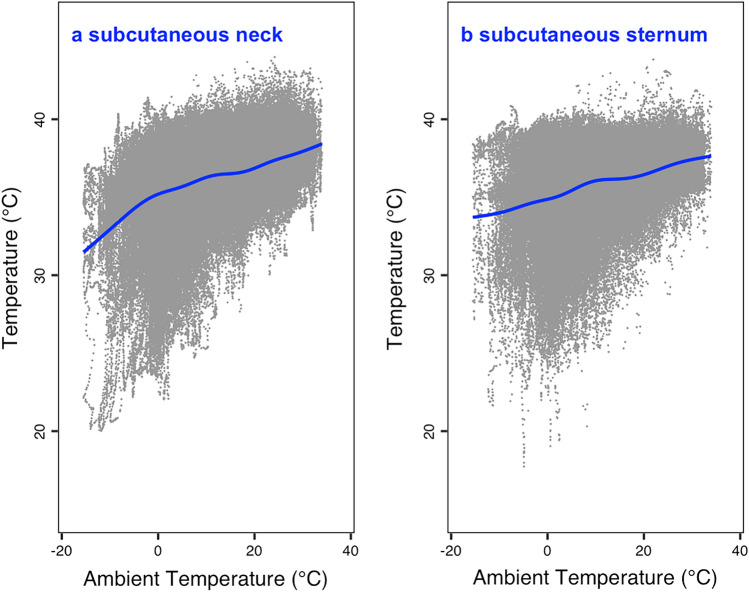
Subcutaneous temperature as a function of T_a_ in wild boar. Temperature loggers measured T_b_ at 12 min intervals and were either fixed to the lateral neck (**a**) next to the sternum (**b**). GAM models (blue lines) indicated that subcutaneous temperatures decreased with T_a_ (neck: edf (wiggliness) = 8.85, residual df = 8.99, *F* = 6281, *P* < 0.0001***; sternum: edf (wiggliness) = 8.54, residual df = 8.94, *F* = 1285, *P* < 0.0001***). Partial residual plots corrected for the effects of random differences between individuals. All models were also corrected for initial autocorrelation (> 0.9)

As T_a_ decreased, floating-intraperitoneal-temperature stayed fairly stable around 38 °C (Fig. 3). In loggers fixed to the peritoneal wall, however, temperature dropped to a minimum of ~ 32 °C around a T_a_ of ~ 0 °C. Below 0 °C, temperature at the peritoneal wall increased again. Subcutaneous temperature both at the neck and the sternum decreased to ~ 25 °C as T_a_ dropped to −15 °C (Fig. 4). The GAM models showed an almost identical decline of both temperatures (Fig. 4). Again, minimum subcutaneous temperature increased as T_a_ fell below ~ 0 °C (Fig. 4). Overall, especially at low T_a_, there was a temperature gradient with intraperitoneal-floating temperature greater than wall temperature, which was in turn greater than subcutaneous temperature.

**Fig. 5:**
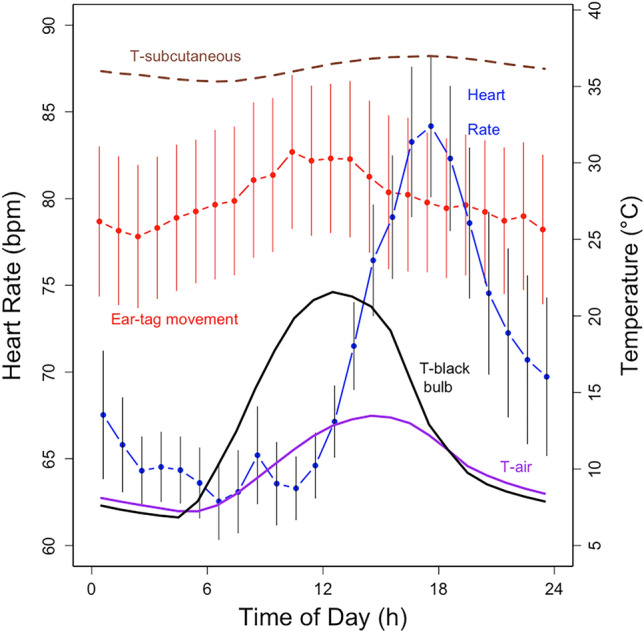
Hourly means of heart rate (blue), ear-tag movements (red; scale not shown) as well as subcutaneous T_b_ (brown) as function of daytime in female wild boar under natural conditions in Austria. Further, ambient (purple) and black-bulb temperatures (black) are shown. The SEM on the mean ear-tag movement and the heat rates indicates the variation among 13 animals, the number of time points of each animal varies. This graph is meant to illustrate the daily rhythm of each variable

Mean subcutaneous temperature also showed a daily rhythm that peaked together with mean heart rate in the afternoon (Fig. 5).

## Discussion

### Heart rate as a proxy

Since each heart beat transports oxygen to tissues, it positively correlates with metabolic rate (MR) under most circumstances (Green 2011). Simultaneous measurements of both variables are rare and usually restricted to small species, but at any physiological state their relation is typically linear (e.g., Butler et al. 2004; Currie et al. 2014; Green 2011). However, here we do not aim to calibrate heart rate, we merely assume that effects of T_a_ on heart rate equally influence MR.

The average heart rate was only about three times that of minimum heart rate. Hence, the wild boar females in this study, which had ample food in addition to their natural forage, were far from maxima observed in mammals (i.e., limits of existence), which can reach up to 12 times basal metabolic rate (Mellish et al. 2000). We also consider energy costs for minimum heart rate during thermoregulation to be only minor (Fig. 2). The missing correlation between “activity” and heart rate suggests that movements of the ears may be a proxy of general alertness but cause no measurable change in energy expenditure.

The only previous observations on thermoregulation in adult wild boar were measurements of MR at various T_a_ in different age-groups (Jezierski and Myrcha 1975). The overall picture obtained was very similar to our observations, with increases below 0 °C (in 11 month old boar) and above + 6 °C (here: + 7 °C), but measurements of adults in or below the TNZ seemed to be missing (Jezierski and Myrcha 1975).

### Responses to low T_a_

Generally, wild boar benefit from their large body size and their near spherical shape, which minimizes the area of heat loss. Vetter et al. (2015) found that body mass among adults correlates negatively with long-term regional mean winter temperatures across Europe. Wild boar in the coldest region were about 30 – 40 kg heavier than in the warmest region. These differences in the body size contribute to the static advantages of wild boar in the cold.

The observed shift in the TNZ from summer to winter was likely due to improved thermal conductance. The obvious, but theoretical, part of this change was the development of a denser winter fur. In winter, the increase in the heart rate in the cold below the TNZ was less than 30% per 10 °C T_a_ decline. This relatively small increase in the heart rate during cold exposure places wild boar in the realm of arctic animals, such as the polar bear, whereas tropical mammals raise their energy expenditure several fold (Heldmaier et al. 2013). We suggest that this resistance is largely due to the development of low peripheral temperatures (Fig. 4), i.e., a pronounced body shell. Low peripheral temperatures decrease the body surface to environment gradient and thus limit heat loss. Temperature gradients in the body belong to the most important adaptations to life in the cold (Blix 2016). The ability to control surface temperature and to regulate heat loss was previously shown to increase with body size (Phillips and Heath 1995). Thus, it is not surprising that it occurs in the wild boar. The fact that these gradients are reduced again at even lower T_a_, maybe due to the avoidance of adverse effects of low T_a_ on the skin, underlines that these gradients result from active regulation rather than an unavoidable cooling of peripheral tissues.

In this context, it should be noted that clades ancestral to the *Suidae* lost the heat generating protein UCP1 in non-shivering thermogenesis (NST) about 40 million years ago (Fyda et al. 2020), as it was lost in many mammalian lineages. UCP1 has been mainly abandoned in mammals of large body size, strong increases in body mass followed each loss of UCP1, and NST capacity is inversely related to body size (Gaudry et al. 2017). Although functional UCP1 was retained by some large mammals (Gaudry et al. 2017), we suggest that a decrease in the role of NST in large animals was partly due to their ability to form large core–shell gradients. Indeed, this is the first time that we can provide a functional explanation for the link between UCP1 loss and large body mass.

One might be tempted to think that in wild boars NST, in the absence of UCP1, plays no role. However, another type of NST, driven by the SERCA pump in muscles, enables juvenile wild boars to generate heat, while shivering is diminishing (Nowack et al. 2019). This type of NST is also present in pigs, the domestic form of wild boar, and seems to be widespread even within adult mammals (Berthon et al. 1994; Nowack et al. 2017). Thus, NST may have contributed to the T_a_ induced thermogenesis observed here.

### Responses to high T_a_s

It seems that energy expenses for heat-avoiding thermoregulatory behavior, that actually cut into energy expenses for foraging, occur in wild boar under heat stress that is more severe than what our animals experienced. The observed response of heart rate to high T_a_ was weak in wild boar across all seasons (Fig. 2). It seems that this very moderate rise in the heart rate was largely due to the choice of a forest as the preferred habitat. This choice of shady areas avoids extreme temperatures that occur in open habitats (for example, see black-bulb temperature in Fig. 5). Wild boar largely lack functional sweat glands for evaporative cooling (Renaudeau et al. 2006) and were seen panting in the field only under severe heat stress (C.B. pers. observation). Only when the hypothalamic temperature is elevated to ~ 39 °C do pigs engage in panting (Baldwin and Ingram 1968). Generally, wild boar and pigs react to changes in temperature, radiation, humidity or wind by adjusting their behavior (Bracke 2011; Olczak et al. 2015). In the heat, pigs and wild boar avoid close contact to conspecifics and particularly use wallowing in mud or other wet substrates to cool and prevent hyperthermia (Bracke 2011; Olczak et al. 2015). Also, it has been argued that wild boars are mainly nocturnal (but see Fig. 5) to lower costs for thermoregulation (e.g., Brivio et al. 2017; Johann et al. 2020).

Low costs for thermoregulation in the heat are also supported by the passive daily rewarming of subcutaneous temperature. Wild boar make extensive use of basking in the sun (Ruf et al. 2021). On days with above average solar radiation, they passively warm peripheral tissues diurnally and thereby save 10% of total energy (Ruf et al. 2021). Arguably, it is thus their diurnality, which allows wild boar to establish low body-to-environment gradients cheaply. Although increased activity and high heart rates during the day (Fig. 5) were probably facilitated by our feeding in the afternoon. We believe observations of nocturnal activity in wild boar are mainly due to their avoidance of human activities, namely hunting (e.g., Brivio et al. 2017; Johann et al. 2020).

### General conclusions

These findings point to the importance of daily rhythms (Fig. 5). It is the reliance on the repeatability of daily cycles, especially of rhythms in subcutaneous temperature that enable wild boar to build large core–shell gradients, which serve to lower heat loss in the cold. Taken together, it seems that the resulting low costs during thermoregulation in the cold act together with effective measures to counteract heat by behavioral strategies in the warm. We argue it is predominantly this ability which allowed wild boar to inhabit most climatically diverse areas (Lewis et al. 2017). We would not be surprised if wild boar showed only small responses to increasing global climate change. However, wild boar are omnivorous, and increasing drought might lead to lower food availability, since digging up roots and insects from below ground seems hardly feasible (Ruf et al. 2021).

## Data Availability

Upon publication of this article, data will be made available from the University of Vienna PHAIDRA data repository (https://www.vetmeduni.ac.at/en/bibliothek/infoservice/phaidra/).
